# Fatigue, Physical Disability and Self-Efficacy as Predictors of the Acceptance of Illness and Health-Related Quality of Life in Patients with Multiple Sclerosis

**DOI:** 10.3390/ijerph182413237

**Published:** 2021-12-15

**Authors:** Joanna Dymecka, Rafał Gerymski, Rafał Tataruch, Mariola Bidzan

**Affiliations:** 1 Department of Health Psychology and Quality of Life, Institute of Psychology, Opole University, 45-040 Opole, Poland; jdymecka@uni.opole.pl; 2 Faculty of Physical Education and Physiotherapy, Opole University of Technology, 45-758 Opole, Poland; r.tataruch@po.edu.pl; 3 Department of Clinical and Health Psychology, Institute of Psychology, University of Gdansk, 80-309 Gdansk, Poland; mariola.bidzan@ug.edu.pl

**Keywords:** multiple sclerosis, fatigue, disability, self-efficacy, acceptance of illness, quality of life

## Abstract

Multiple sclerosis (MS) is a chronic progressive demyelinating disease of the central nervous system that leads to permanent disability and many neurological symptoms, making everyday functioning difficult. The predictors of the acceptance of illness and the health-related quality of life in people with MS include the degree of disability, neurological symptoms and psychosocial factors, such as personal resources. The aim of our study is to determine the relationships among disability, fatigue, self-efficacy, acceptance of illness and quality of life. The study group consisted of 137 people diagnosed with multiple sclerosis—73 women and 64 men. EDSS, GNDS, LSES, AIS and MSIS-29 were used in the present study. The results show that all tested variables were significantly correlated with each other. Disability and fatigue were significant predictors of both the physical and psychological aspects of patients’ quality of life. Self-efficacy was a significant predictor of both the acceptance of illness and the psychological aspect of patients’ quality of life. Based on the current research study, it can be concluded that factors of a biomedical nature explain other aspects of struggling with the disease, rather than psychological resources.

## 1. Introduction

Multiple sclerosis (MS) is a chronic inflammatory demyelinating disease of the central nervous system. Due to its prevalence, MS is the leading cause of non-traumatic neurological disability in young and middle-aged people. MS is a disease with a varied clinical course. In some people, it is mild or asymptomatic; however, there are also forms of the illness that are rapid and lead to significant disability in a short time. There are four main forms of multiple sclerosis, namely, relapsing–remitting, primary progressive, secondary progressive and relapsing–progressive. In addition to the fact that the differentiation of MS types is related to the frequency of relapses and the rate of progression of neurological failure, it is also associated with the symptoms that occur. In patients with multiple sclerosis, all symptoms associated with damage to the central nervous system may appear [1,2,3].

MS has serious negative effects on patients’ physical and psychosocial functioning. People with MS struggle with uncertainty about their future, unpleasant and unpredictable signs and symptoms and the side effects of treatments. The diagnosis of an illness such as MS has many negative consequences, affecting life goals, employment, social relationships and activities of daily living. In order to function in the new reality marked by MS, the patient must develop appropriate coping strategies [4]. Neurological dysfunctions that cause disability, such as MS, are associated with significant difficulties in adapting to them. The degree of acceptance of illness is a determinant of the patient’s functioning. Such acceptance is associated with less discomfort and low intensity of negative emotions related to the disease. Acceptance of MS can be considered an indicator of adaptation to the limitations imposed by it [5].

Multiple sclerosis is a disease that affects patients’ health-related quality of life (HRQoL) [6]. Studies show that the HRQoL of people with MS is significantly lower than that of healthy people [7,8,9,10]. People with MS have lower quality of life than healthy people in terms of the general level and also its dimensions [11]. Moreover, life satisfaction in people with MS is significantly lower than that in people with other chronic diseases such as enteritis, rheumatoid arthritis, epilepsy, diabetes, or cardiovascular diseases [12,13,14,15]. Multiple sclerosis is a chronic disease leading to permanent disability; therefore, physical disability was initially indicated as one of the most important predictors of quality of life in this group of patients [16]. A strong negative correlation between the degree of physical disability of MS patients and their quality of life has been demonstrated in many studies [5,8,16,17,18]. It has been shown that people who can move independently assess their quality of life higher than people who need crutches or a wheelchair [5]. Some studies show that physical disability has a more negative impact on HRQoL than mental problems [19]. On the other hand, disability is mainly related to the physical aspect of MS patients’ quality of life [20]. However, most researchers indicate that the relationship between physical disability and quality of life is not direct [21]. Therefore, researchers are looking for other factors that may affect the physical and mental HRQoL and adaptation to MS.

One of the most common and bothersome symptoms of multiple sclerosis is fatigue. It is defined as a sense of exhaustion associated with the need to rest, or a subjective feeling of lack of physical or mental energy that interferes with daily activities. Fatigue in multiple sclerosis can manifest itself as fatigue after exercise, weakness, fatigue even while resting and exacerbation of other symptoms. It may be the first symptom of MS before diagnosis [22,23]. The prevalence of fatigue in people with MS is estimated to be from 50% to 90%, making it one of the most frequently reported symptoms of the disease [24]. Many studies have shown that fatigue is an important factor affecting functioning in MS, acceptance of MS and quality of life [5,25,26,27], even in the initial stages [13]. Patients at any stage of MS, even those with mild disability, may experience significant fatigue, which is considered, by 40–50%, as the most severe symptom of the disease [13]—more burdensome than pain or even physical disability [28]. Therefore, it is important to analyze the importance of fatigue for the quality of life of MS patients and their acceptance of illness.

Moreover, many researchers indicate that emotional and social problems have a similar impact on the acceptance of illness and HRQoL of MS patients [29,30]. Some psychosocial factors, such as stress management and personal resources, can influence the quality of life in MS to a greater extent than some biomedical variables [13]. Many studies show that self-efficacy is a particularly important resource in coping with multiple sclerosis. This is an individual’s belief in their capacity to execute behaviors necessary to produce specific performance attainments. It is also the belief that a person can carry out a specific action or achieve set goals. It is a general psychological mechanism that can influence an individual’s activity and effectiveness [31]. The sense of self-efficacy can also be understood as an individual’s belief that they can cope with a given activity or task even in new, unpredictable, difficult and stressful conditions [32]. Psychosocial resources, such as self-efficacy, have a significant impact on the functioning of people with chronic dysfunctions. They affect the healing processes and the individual’s motivation to improve their health situation. Self-efficacy influences the perception of one’s health and the impact of health on one’s life [33]. Therefore, self-efficacy can be treated as a predictor of how an individual with MS adapts to it [34,35]. It has been shown that self-efficacy plays an important role in MS patients’ engaging in social activity and in controlling negative thoughts [36]. It is also related to their HRQoL [13,37,38].

Based on the presented introduction, we believe that physical disability, by limiting mobility and independence, may affect everyday functioning and, above all, the physical aspect of HRQoL. Fatigue, as the most common symptom of MS and, at the same time, an invisible symptom, can significantly limit activity and participation in social life, thus affecting HRQoL. On the other hand, the sense of self-efficacy seems to be one of the most important resources influencing coping with multiple sclerosis, thus the acceptance thereof. Based on the above studies, it can be concluded that physical disability, fatigue and self-efficacy may be significant predictors of MS patients’ acceptance of illness and the physical and mental aspects of HRQoL. However, multiple sclerosis is an unpredictable disease and each individual patient may experience it differently [3,4,5,12] Therefore, the present study aims to (1) verify whether the studied sample of patients is a homogeneous group and (2) determine the relationship between disability, fatigue, self-efficacy, acceptance of illness and the HRQoL of Polish multiple sclerosis patients.

## 2. Materials and Methods

### 2.1. Participants and Procedure

We decided to verify the aforementioned conclusions in a cross-sectional correlational study. A total of 137 people diagnosed with multiple sclerosis participated in the study—73 women and 64 men. The youngest of the examined persons was 18 years old and the oldest was 73. The average age of the examined patients with MS was 46.47 years (*SD* = 12.59). They were patients staying at rehabilitation camps at the Rehabilitation Centre for People with Multiple Sclerosis in Borne Sulinowo (Poland) and members of foundations and associations providing help to people with MS—the Association of Patients with Multiple Sclerosis in Głogów (Poland) and the “Twardziele” group (Tricity, Poland). Patients with cognitive deficits that made it difficult to understand psychological questionnaires (i.e., patients who scored more than 3 points on the Cognitive Disorders subscale of the GNDS questionnaire) were excluded from the study. The mean duration of MS in the study sample was 14.61 years (*SD* = 8.31). Disease-modifying therapy was used by 62.04% of the studied patients and 37.96% had never had access to this type of therapy. Only about 8% of patients had participated in drug programs for more than 5 years. For more detailed information, see Table 1.

The examination was carried out during one meeting with the patient. The meeting had no time limit and its duration was adjusted to the psychophysical abilities of the patient. The full psychological examinations lasted from about 45 min to 2 h. Before participating in the study, the patients were asked to give their full consent. They were informed about the purpose of the research study, its anonymity and that all data would have been used for research purposes only. The study consisted of the patients completing a set of questionnaires, which were always given in the same order. The study was approved by the Ethics Committee at the Institute of Psychology of the University of Gdańsk (No. 19/06/2015). All respondents gave their consent to participate in the study.

### 2.2. Measures

Fatigue was measured with Guy Hospital’s Neurological Disability Scale (GNDS) [39] in its Polish adaptation [40]. It consists of 12 subscales concerning individual areas of functioning, measured with 72 questions on a dichotomous nominal scale (where 1—Yes; 0—No) as follows: cognitive disorders, mood disorders, problems with eyesight, speech, swallowing, upper limb functioning, lower limb function, functioning of the bladder and intestines, problems with sexual functioning and fatigue. GNDS measures fatigue with 5 questions, concerning feeling tired during the last month, feeling tired most days, the effect of fatigue on performing certain daily activities, all daily activities and whether fatigue was keeping the patient from doing any physical activity. A high score on a particular scale indicates a high level of the perceived symptoms. We decided to use this as a measure of fatigue, as it was specifically designed for people with multiple sclerosis and shows good reliability (in the present study, Cronbach’s alpha = 0.73).

Physical disability was operationalised with the Expanded Disability Status Scale (EDSS) [41]. It is widely used as a method of evaluating the degree of neurologic impairment among MS patients. EDSS ranges from 1 to 10 in increments of 0.5, for a total of 20 possible degrees of disability. The higher the score on the scale, the greater the disability, where 1—no disability; 5—disability severe enough to impair full daily activities; and 10—death due to MS. Due to the nature of the scale, standard reliability coefficients such as Cronbach’s alpha cannot be calculated for it.

Self-efficacy was measured with the Liverpool Self-efficacy Scale (LSES) [42] in its Polish adaptation [43]. LSES is an 11-item scale, which is composed of two subscales, i.e., control (6 items) and personal agency (5 items). Participants may express their opinion on a 4-point scale, where 1 is “I strongly agree”, 2 is “I agree”, 3 “I do not agree” and 4 is “I definitely disagree”. Some items contain reverse scores. The higher the score, the higher the patients’ self-efficacy. The reliability of the original version of the questionnaire was determined using Cronbach’s alpha coefficient, which was 0.81.

The Acceptance of Illness Scale (AIS) [44] in its Polish adaptation [45], was used to assess patients’ adaptation to limitations caused by MS. It contains 8 statements describing consequences of ill health. In each statement, the respondent describes his or her current state on a five-point scale (1—I strongly agree; 5—I strongly disagree). A low result indicates lack of acceptance of the illness and a strong sense of psychological discomfort, whereas a high result indicates acceptance of the disease and a lack of negative emotions related to it. The reliability of the Polish version of the scale is satisfactory (in the present study, Cronbach’s alpha = 0.85).

The physical and psychological impact of MS was measured with the Multiple Sclerosis Impact Scale 29 (MSIS-29) [46] in its Polish adaptation [47]. It is one of several scales specific to MS to measure quality of life in this group of patients. It consists of 29 questions, 20 of which are related to the physical state and 9 to the mental state. The participant responds to each of the test items on a 5-point Likert scale, ranging from 1 (Not at all) to 5 (Extremely). The higher the score, the higher the impact of MS on a given sphere of functioning. The reliability of the Polish version of the scale is acceptable (in the present study, Cronbach’s alpha = 0.83–0.87).

### 2.3. Data Analyses

The *t*-test and ANOVA analysis with Tukey’s honest significant difference (HDS) post hoc test were used to verify the significance of the differences between studied groups. The Pearson *r* correlation and multivariate regression were used to estimate the relationships between selected variables. the analyses were conducted using IBM SPSS 24 (IBM Polska Sp. z o.o., Warsaw, Poland). All statistical tests were two-tailed and the significance level was set to α = 0.05.

## 3. Results

### 3.1. Group Homogeneity Analysis

In the first step of the statistical analyses, it was verified whether the studied group of MS patients was a homogeneous sample. Gender comparisons were made using the *t*-test for independent groups. The analysis did not show any significant gender differences in any of the tested variables—fatigue, physical disability, self-efficacy, acceptance of illness, or physical and psychological impact of MS. For more detailed information, see Table 2.

In the next step, it was verified whether the type of MS was a significant grouping variable for the studied dependent variables. For this, ANOVA with Tukey’s HSD post hoc test was performed. The analysis showed that people with the relapsing–remitting form of MS (*n* = 43) had significantly lower results of physical disability and physical impact of the MS than the groups of patients with the primary (*n* = 22) and secondary progressive (*n* = 31) forms of MS. This means that the severity of disability and the influence of MS on functioning in the physical sphere was greater in patients with primary and secondary progressive forms of MS than in patients with relapsing–remitting MS. The other differences were not statistically significant. Detailed information is provided in Table 3.

### 3.2. Relationship between the Studied Variables

The Pearson *r* correlation was used in order to verify the relationships between the studied variables. All the studied relationships were statistically significant. Fatigue was positively associated with the results of physical disability and physical and psychological impact of MS and negatively with the results of self-efficacy and acceptance of illness. This means that, with the increase in fatigue in the study sample, there was an increase in physical disability scores and the impact of MS on selected spheres of functioning. The increase in fatigue scores was also associated with decreased self-efficacy and acceptance of illness. Physical disability was positively associated with the physical and psychological impact of MS and negatively with the results of self-efficacy and acceptance of illness. The relationships of self-efficacy and acceptance of illness with the physical and psychological impact of MS were negative. More detailed information is presented in Table 4.

In the last step, it was decided to verify which of the studied variables would be statistically significant predictors of acceptance of illness and the impact of MS on the physical and psychological spheres. For this purpose, three multiple regression analyses were used. They showed that only self-efficacy was a significant predictor of acceptance of illness and the tested model explained 33% of the dependent variable’s variance. Fatigue, physical disability and psychological impact of MS were statistically significant predictors of the physical impact of MS and explained 65% of its variance. For the psychological impact of MS, significant predictors were: fatigue, physical disability, self-efficacy and physical impact of MS. This model accounted for 55% of the variance of the dependent variable results. For more information, see Table 5.

## 4. Discussion

Multiple sclerosis is a disease with many signs and symptoms that can lead to permanent disability. This study analyzed the relationships between physical disability, fatigue, self-efficacy, acceptance of illness and the physical and mental dimensions of health-related quality of life (HRQoL). It was found that all variables were significantly correlated with each other and that disability, fatigue and self-efficacy were significant predictors of MS patients’ functioning.

In our study, there was a statistically significant, moderate and negative correlation between acceptance of illness and motor impairment. This means that the greater the disability in patients with MS, the worse their acceptance of illness. There was also a statistically significant positive correlation between HRQoL and degree of motor impairment. Although physical disability was significantly correlated with both acceptance of illness and HRQoL, it was shown that it is only a predictor of HRQoL, including primarily its physical aspect. For the physical component of HRQoL, disability explained a significant percentage of the variance of the dependent variable. Physical disability, which is associated with problems in everyday functioning and dependence on others, makes it more difficult to live with the disease, thus more difficult to accept [48]. A strong negative correlation between the degree of physical disability and quality of life has been demonstrated in many studies [5,8,16,18,28,49,50]. Some studies show that physical disability has a more negative impact on HRQoL than mental problems [19]. However, others indicate that the relationship between EDSS and HRQoL is not simple. Some researchers believe that disability is a better predictor of HRQoL at the onset of the disease than in the later stages, when patients adapt to the disease [18]. However, others believe that HRQoL of people with MS is influenced not only by EDSS, but also by the interaction of physical, psychological and social factors [19] and disability is only one of many predictors [51]. Moreover, increased disability may negatively affect mental functioning, which affects the quality of life of patients with MS [52]. Some studies show that most aspects of HRQoL are only weakly related to disability [12,51,53]. However, the results of the current research study contradict those that indicate that physical disability has only a slight relationship with HRQoL. The relationship between disability and HRQoL in the present study is strong and the EDSS score was a significant predictor of HRQoL, especially its physical aspect. The results of our research study may also indicate that it is important to analyze various aspects of quality of life, as each of them is influenced in a variety of ways by the clinical features of the MS.

In addition to mobility problems, disability in multiple sclerosis may affect other areas of functioning. MS also includes invisible symptoms such as fatigue, which, as shown in the present study, is a symptom significantly associated with both acceptance of illness and two aspects of MS patients’ HRQoL. Similar to physical disability, fatigue was a significant predictor of both dimensions of quality of life. Contrary to the disability measured by the EDSS, no significant asymmetry in explaining the physical and mental dimensions of HRQoL was noticed. In our study, fatigue was a symptom that significantly predicted both aspects of the MS patients’ HRQoL. The relationship between fatigue, acceptance of illness and quality of life has been analyzed in many studies. Fatigue, being one of the most common and bothersome symptoms, is an important factor influencing the physical and mental HRQol even in patients in the early stages of MS [13,54]. It has been indicated that 40–50% of patients identify fatigue as the most severe symptom of the disease, more burdensome than pain or even physical disability [28]. Fatigue is the main cause of the inability to work and early retirement [55,56]. It can lead to an increase in existing disability and negatively affect the results of rehabilitation [23]. It is a symptom that influences daily activity [57], having a negative impact on the quality of life [58], regardless of physical disability or psychological problems such as depression [12].

In the present study, in addition to the factors related to the course of MS, the importance of self-efficacy was also analyzed. It is shown that self-efficacy was associated with both disability and fatigue. We also found strong correlation among self-efficacy, acceptance of illness and both dimensions of HRQoL in patients with MS. In our study, self-efficacy was also a significant predictor of acceptance of illness and the psychological aspect of HRQoL of MS patients. Self-efficacy is a variable that is very important in the adaptation to chronic dysfunctions. Self-efficacy may play an important role in an individual’s adaptation to the wide range of symptoms of MS and the uncertainty associated with the illness [37,59]. It has been indicated that it may play a more important role than biomedical variables [60,61], which was confirmed in the present study. In our study, only self-efficacy turned out to be a significant predictor of the acceptance of illness. A relationship between self-efficacy and acceptance of illness has also been demonstrated among other groups of patients. In people with type 1 diabetes and rheumatoid arthritis, self-efficacy was conducive to illness acceptance [62]. Self-efficacy affects coping with the illness because it is related to the human motivation to act [63]. Low self-efficacy may lower the motivation to deal with the MS. According to Kościelak [64], people who have little conviction about their effectiveness at the time of disease emergence or its exacerbation are more likely to give up activities that could improve their health. People with a strong belief in their own effectiveness believe that they can cope with a given situation even in difficult and unfavorable circumstances, while people with a low sense of self-efficacy are not sure of their abilities, which is associated with low motivation to act and little willingness to take up challenges [31]. The study also showed that self-efficacy is a predictor of the mental aspect of quality of life. Self-efficacy is usually more closely related to the mental aspect of quality of life than with the physical aspect. A strong belief in illness control and one’s coping skills may reduce the negative impact of multiple sclerosis on the quality of life, which has also been shown in other studies [13,37,38,65]. Self-efficacy was also positively associated with quality of life in a study of 786 people with MS [66] and another study found that self-efficacy was a predictor of mental well-being in people with MS [36], which is consistent with our research study.

Unfortunately, as any other research project, this one is not free from limitations. First, our results were based on a cross-sectional study. Longitudinal studies should be conducted to verify the predictive role of the studied variables in the HRQoL of MS patients’. Our paper discusses the obtained results based on the theoretical background, without any definite causal conclusions. Longitudinal research work is needed to determine causality and the predictive value of the tested variables. Second, the sample was treated in the analyses as a homogeneous group. However, this does not mean that all respondents were very similar to each other. The omission of testing for other biomedical variables such as physical activity is a major limitation of the presented research project and should be taken into account in future studies. Other studies suggest that there is a significant relationship between self-efficacy and physical activity in people with MS [37,65,67]. Those studies found that self-efficacy influenced the physical activity of the individual and the expected effects of this activity. People who were confident in their ability to engage in physical activity and who believed they could overcome obstacles to undertaking activity were more physically active than those with low self-efficacy. People with higher self-efficacy expected greater effects and believed that the consequences of exercise would be beneficial [68]. Self-efficacy was also an important predictor of the ability of people with MS to move independently [69]. A relationship has also been demonstrated between self-efficacy and objective gait performance [70]. This proves the significant importance of self-efficacy for the mobility of people with MS. Moreover, according to Bandura’s socio-cognitive model, the physical activity of an individual could influence their self-efficacy, which may indirectly influence the quality of life through health-related variables, including functional limitations. Physically active people have a greater sense of self-efficacy and better endurance, which is associated with a higher quality of life [65]. Based on self-efficacy, it is also possible to predict mood control, social activity and self-esteem [36,42]; therefore, self-efficacy can be considered a very important variable in the adaptation to and acceptance of multiple sclerosis and other chronic diseases [6,43,71,72].

## 5. Conclusions

Multiple sclerosis is a disease associated with significant challenges. It is a problem that can cause significant disability associated with many neurological symptoms. The present study shows that disability and fatigue are important predictors of both the physical and mental aspects of quality of life. Disability explained the most variance for the physical aspect of HRQoL. However, both of these factors were not significant predictors of acceptance of illness. It can be concluded that psychological factors are probably more important for accepting MS. One such factor is self-efficacy, which, in our study, was a significant predictor of both the acceptance of illness and the mental aspect of the health-related quality of life in MS patients. Based on the current research, it can be concluded that factors of a biomedical nature explain different aspects of struggling with MS compared with psychological resources. The current study also shows that personal resources, such as self-efficacy, are important for accepting MS and its consequences. It is easier for a person who believes in their abilities and who is motivated to exercise and rehabilitate to deal with the limitations associated with the disease.

## Figures and Tables

**Table 1:** Characteristics of the studied sample.

	*M*	*SD*	*Min*	*Max*
Age	46.47	12.59	18.00	73.00
Age of the diagnosis	33.94	10.65	15.00	61.00
Illness duration (in years)	14.61	8.31	<1.00	42.00
	*n*		%	
Gender				
Women	73		53.28%	
Men	64		46.72%	
Education				
Elementary school	2		1.46%	
Vocational	25		18.25%	
High school	58		42.34%	
University—Bachelor’s Degree	13		9.49%	
University—Master’s Degree	38		27.74%	
No data	1		0.73%	
Type of Multiple Sclerosis				
Relapsing–remitting	43		31.39%	
Primary progressive	22		16.06%	
Secondary progressive	31		22.63%	
Progressive–relapsing	8		5.84%	
Undefined	33		24.09%	

**Table 2:** Results of *t*-test comparisons.

	Women		Men		*t* (*df*)	*p*	*d_Cohen_*
	*M*	*SD*	*M*	*SD*			
Fatigue	3.10	1.73	2.59	1.70	1.72 (133)	0.087	0.30
Physical disability	4.29	2.19	4.90	1.97	−1.71 (135)	0.090	0.29
Self-efficacy	27.26	6.17	28.09	5.56	−0.83 (135)	0.410	0.14
Acceptance of illness	24.58	8.24	23.77	8.94	0.55 (135)	0.582	0.09
Physical impact of MS	51.59	20.02	51.66	18.68	−0.02 (135)	0.984	<0.01
Psychological impact of MS	24.60	9.73	22.77	9.16	1.13 (135)	0.259	0.19

**Table 3:** Results of the ANOVA comparisons.

	Grouping Variable: Form of the Multiple Sclerosis			
	*F* (*df*_1_; *df*_2_)	*p*	η ² *_p_*	Tukey’s HSD
Fatigue	1.97 (4; 130)	0.103	0.06	*–*
Physical disability	4.89 (4; 132)	0.001	0.13	PP > R-R; SP > R-R
Self-efficacy	0.23 (4; 132)	0.921	<0.01	*–*
Acceptance of illness	0.24 (4; 132)	0.920	<0.01	*–*
Physical impact of MS	4.85 (4; 132)	0.001	0.13	PP > R-R; SP > R-R
Psychological impact of MS	1.07 (4; 132)	0.374	0.03	*–*

Note: PP—primary progressive; SP—secondary progressive; R-R—relapsing–remitting.

**Table 4:** Results of the Pearson r correlation.

	M	SD	1.	2.	3.	4.	5.	6.
Fatigue	2.86	1.73	-					
2. Physical disability	4.57	2.10	0.19 *	-				
3. Self-efficacy	27.65	5.88	−0.37 ***	−0.33 ***	-			
4. Acceptance of illness	24.20	8.55	−0.33 ***	−0.31 ***	0.53 ***	-		
5. Physical impact of MS	51.62	19.33	0.43 ***	0.65 ***	−0.44 ***	−0.40 ***	-	
6. Psychological impact of MS	23.74	9.48	0.47 ***	0.20 *	−0.56 ***	−0.42 ***	0.59 ***	-

Note: * *p* < 0.5; *** *p* < 0.001.

**Table 5:** Results of three separate multiple regression analyses.

DV	Predictors	*Beta*	*SE_Beta_*	*t* (129)	*p*	Model Summary		
						*F* (5; 129)	*p*	*R* ^2^
Acceptance of illness	Fatigue	−0.09	0.08	−1.07	0.287	12.67	<0.001	0.33
	Physical disability	−0.11	0.10	−1.08	0.283			
	Self-efficacy	0.37	0.09	3.98	<0.001			
	Physical impact of MS	−0.06	0.12	−0.45	0.650			
	Psychological impact of MS	−0.12	0.11	−1.12	0.263			
Physical impact of MS	Fatigue	0.13	0.06	2.17	0.032	48.65	<0.001	0.65
	Physical disability	0.55	0.06	9.84	<0.001			
	Self-efficacy	0.04	0.07	0.56	0.575			
	Acceptance of illness	−0.03	0.06	−0.45	0.650			
	Psychological impact of MS	0.42	0.07	6.24	<0.001			
Psychological impact of MS	Fatigue	0.14	0.07	2.07	0.041	31.02	<0.001	0.55
	Physical disability	−0.32	0.08	−4.06	<0.001			
	Self-efficacy	−0.33	0.07	−4.49	<0.001			
	Acceptance of illness	−0.08	0.07	−1.12	0.263			
	Physical impact of MS	0.55	0.09	6.24	<0.001			

## Data Availability

The data can be made available from the corresponding author upon reasonable request.

## References

[1] Baecher-Allan C., Kaskow B.J., Weiner H.L. (2018). Multiple Sclerosis: Mechanisms and Immunotherapy. Neuron.

[2] Cotsapas C., Mitrovic M., Hafler D. (2018). Multiple sclerosis. Handb. Clin. Neurol..

[3] Klineova S., Lublin F.D. (2018). Clinical Course of Multiple Sclerosis. Cold Spring Harb. Perspect. Med..

[4] Dymecka J., Bidzan M., Bieleninik Ł., Szulman-Wardal A. (2015). Radzenie sobie z własną chorobą u osób ze stwardnieniem rozsianym [Coping with a disease in people with Multiple Sclerosis]. Niepełnosprawność Ruchowa w Ujęciu Biopsychospołecznym. Wyzwania Diagnozy, Rehabilitacji i Terapii [Physical Disability in the Biopsychosocial Approach. Challenges of Diagnosis, Rehabilitation and Therapy].

[5] Dymecka J., Bidzan M. (2018). Biomedical Variables and Adaptation to Disease and Health-Related Quality of Life in Polish Patients with MS. Int. J. Environ. Res. Public Health.

[6] Dymecka J., Krok D., Dymecka J. (2020). Jakość życia uwarunkowana stanem zdrowia u osób ze stwardnieniem rozsianym [Health-related quality of life in people with multiple sclerosis]. Jakość Życia a Zdrowie: Uwarunkowania i Konsekwencje [Quality of Life and Health: Conditions and Consequences].

[7] Amtmann D., Bamer A.M., Kim J., Chung H., Salem R. (2018). People with multiple sclerosis report significantly worse symptoms and health related quality of life than the US general population as measured by PROMIS and NeuroQoL outcome measures. Disabil. Health J..

[8] Beiske A.G., Naess H., Aarseth J.H., Andersen O., Elovaara I., Farkkila M., Hansen H.J., Mellgren S.I., Sandberg-Wollheim M., Sorensen P.S. (2007). Health-related quality of life in secondary progressive multiple sclerosis. Mult. Scler..

[9] Casetta I., Riise T., Wamme Nortvedt M., Economou N.T., De Gennaro R., Fazio P., Cesnik E., Govoni V., Granieri E. (2009). Gender differences in health-related quality of life in multiple sclerosis. Mult. Scler..

[10] Klevan G., Jacobsen C.O., Aarseth J.H., Myhr K.M., Nyland H., Glad S., Lode K., Figved N., Larsen J.P., Farbu E. (2014). Health related quality of life in patients recently diagnosed with multiple sclerosis. Acta Neurol. Scand..

[11] McCabe M.P., Stokes M., McDonald E. (2009). Changes in quality of life and coping among people with multiple sclerosis over a 2 year period. Psychol. Health Med..

[12] Benito-León J., Morales J.M., Rivera-Navarro J., Mitchell A. (2003). A review about the impact of multiple sclerosis on health-related quality of life. Disabil. Rehabil..

[13] Mitchell A.J., Benito-León J., González J.M., Rivera-Navarro J. (2005). Quality of life and its assessment in multiple sclerosis: Integrating physical and psychological components of wellbeing. Lancet Neurol..

[14] Naess H., Beiske A.G., Myhr K.M. (2008). Quality of life among young patients with ischaemic stroke compared with patients with multiple sclerosis. Acta Neurol. Scand..

[15] Rudick R.A., Miller D., Clough J.D., Gragg L.A., Farmer R.G. (1992). Quality of life in multiple sclerosis. Comparison with inflammatory bowel disease and rheumatoid arthritis. Arch. Neurol..

[16] Spain L.A., Tubridy N., Kilpatrick T.J., Adams S.J., Holmes A.C. (2007). Illness perception and health-related quality of life in multiple sclerosis. Acta Neurol. Scand..

[17] Khodaveisi M., Ashtarani F., Mahjub H. (2018). Correlations between Severity of Disease and Quality of Life in Patients with Multiple Sclerosis in Hamadan. Jundishapur J. Chronic Dis. Care.

[18] Miller A., Dishon S. (2006). Health-related quality of life in multiple sclerosis: The impact of disability, gender and employment status. Qual. Life Res..

[19] Tadić D., Dajić V. (2013). Quality of life in patients with multiple sclerosis in Republic of Srpska. Med. Glas.

[20] Effat S., Azzam H., Shalash A., Elkatan S., Elrassas H. (2016). Self-reported quality of life of patients with multiple sclerosis with mild disability. Egypt J. Neurol. Psychiatry Neurosurg..

[21] Ford H.L., Gerry E., Johnson M.H., Tennant A. (2001). Health status and quality of life of people with multiple sclerosis. Disabil. Rehabil..

[22] Hourihan S.J. (2015). Managing fatigue in adults with multiple sclerosis. Nurs. Stand..

[23] Barnett R. (2005). Fatigue. Lancet.

[24] Krupp L. (2006). Fatigue is intrinsic to multiple sclerosis (MS) and is the most commonly reported symptom of the disease. Mult. Scler..

[25] Farran N., Safieddine B.R., Bayram M., Abi Hanna T., Massouh J., AlKhawaja M., Tamim H., Darwish H. (2020). Factors affecting MS patients’ health-related quality of life and measurement challenges in Lebanon and the MENA region. Mult. Scler J. Exp. Transl. Clin..

[26] Strober L.B. (2018). Quality of life and psychological well-being in the early stages of multiple sclerosis (MS): Importance of adopting a biopsychosocial model. Disabil. Health J..

[27] Tabrizi F.M., Radfar M. (2015). Fatigue, Sleep Quality, and Disability in Relation to Quality of Life in Multiple Sclerosis. Int. J. MS Care.

[28] Janardhan V., Bakshi R. (2002). Quality of life in patients with multiple sclerosis: The impact of fatigue and depression. J. Neurol. Sci..

[29] Dymecka J., Gerymski R. (2019). Niepełnosprawność a jakość życia pacjentów ze stwardnieniem rozsianym. Mediacyjna rola zapotrzebowania na wsparcie społeczne [Disability and the quality of life of patients with multiple sclerosis. Mediating role of the need for social support]. Człowiek–Niepełnosprawność–Społeczeństwo Man Disabil. Soc..

[30] Dymecka J., Gerymski R. (2020). Role of resiliency in the relationship between disability and quality of life of people with multiple sclerosis: Mediation analysis. Adv. Psychiatry Neurol..

[31] Bandura A. (1977). Self-efficacy: Toward a unifying theory of behavioral change. Psychol. Rev..

[32] Luszczynska A., Scholz U., Schwarzer R. (2005). The general self-efficacy scale: Multicultural validation studies. J. Psychol..

[33] Rigby S.A., Domenech C., Thornton E.W., Tedman S., Young C.A. (2003). Development and validation of a self-efficacy measure for people with multiple sclerosis: The Multiple Sclerosis Self-efficacy Scale. Mult. Scler..

[34] Amtmann D., Bamer A.M., Cook K.F., Askew R.L., Noonan V.K., Brockway J.A. (2012). University of Washington self-efficacy scale: A new self-efficacy scale for people with disabilities. Arch. Phys. Med. Rehabil..

[35] Schmitt M.M., Goverover Y., Deluca J., Chiaravalloti N. (2014). Self-efficacy as a predictor of self-reported physical, cognitive, and social functioning in multiple sclerosis. Rehabil. Psychol..

[36] Barnwell A.M., Kavanagh D.J. (1997). Prediction of psychological adjustment to multiple sclerosis. Soc. Sci. Med..

[37] Motl R.W., Snook E.M. (2008). Physical activity, self-efficacy, and quality of life in multiple sclerosis. Ann. Behav. Med..

[38] Guicciardi M., Carta M., Pau M., Cocco E. (2019). The Relationships between Physical Activity, Self-Efficacy, and Quality of Life in People with Multiple Sclerosis. Behav. Sci..

[39] Sharrack B., Hughes R.A. (1999). The Guy’s Neurological Disability Scale (GNDS): A new disability measure for multiple sclerosis. Mult. Scler..

[40] Dymecka J., Bidzan M., Rautszko R., Bidzan-Bluma I., Atroszko P. (2017). Skala Niesprawności Neurologicznej Szpitala Guy jako istotne narzędzie do oceny objawów występujących u osób z SM [Guy’s Neurobiological Disability Scale as a significant tool to assess symptoms occurring in patients with MS]. Niepełnosprawność Zagadnienia Probl. Rozw..

[41] Kurtzke J.F. (2015). On the origin of EDSS. Mult. Scler. Relat. Disord..

[42] Airlie J., Baker G.A., Smith S.J., Young C.A. (2001). Measuring the impact of multiple sclerosis on psychosocial functioning: The development of a new self-efficacy scale. Clin. Rehabil..

[43] Dymecka J., Gerymski R., Bidzan M. (2020). Selected psychometric aspects of the Polish version of the Liverpool Self-efficacy Scale. Curr. Issues Personal. Psychol..

[44] Felton B.J., Revenson T.A., Hinrichsen G.A. (1984). Stress and coping in the explanation of psychological adjustment among chronically ill adults. Soc. Sci. Med..

[45] Juczyński Z. (2001). Narzędzia w Promocji i Psychologii Zdrowia [Tools in the Promotion and Psychology of Health].

[46] Hobart J., Lamping D., Fitzpatrick R., Riazi A., Thompson A. (2001). The Multiple Sclerosis Impact Scale (MSIS-29): A new patient-based outcome measure. Brain.

[47] Jamroz-Wiśniewska A., Papuć E., Bartosik-Psujek H., Belniak E., Mitosek-Szewczyk K., Stelmasiak Z. (2007). Analiza walidacyjna wybranych aspektów psychometrycznych polskiej wersji Skali Wpływu Stwardnienia Rozsianego na Jakość Zycia Chorych (MSIS-29) [Validation of selected aspects of psychometry of the Polish version of the Multiple Sclerosis Impact Scale 29 (MSIS-29)]. Neurol. Neurochir. Pol..

[48] Dymecka J., Gerymski R. (2020). Acceptance of illness as a mediator of the relationship between neurological disability and health-related quality of life of people with multiple sclerosis. Neuropsychiatry Neuropsychol..

[49] Fernández-Jiménez E., Arnett P.A. (2015). Impact of neurological impairment, depression, cognitive function and coping on quality of life of people with multiple sclerosis: A relative importance analysis. Mult. Scler..

[50] Talarska D., Brzozowska E. (2003). Jakość życia pacjentów ze stwardnieniem rozsianym [Quality of life of patients with multiple sclerosis]. Neurol. Neurochir. Pol..

[51] Fischer J.S., LaRocca N.G., Miller D.M., Ritvo P.G., Andrews H., Paty D. (1999). Recent developments in the assessment of quality of life in multiple sclerosis (MS). Mult. Scler..

[52] Kikuchi H., Mifune N., Niino M., Kira J., Kohriyama T., Ota K., Tanaka M., Ochi H., Nakane S., Kikuchi S. (2013). Structural equation modeling of factors contributing to quality of life in Japanese patients with multiple sclerosis. BMC Neurol..

[53] Morales-Gonzáles J.M., Benito-León J., Rivera-Navarro J., Mitchell A.J., GEDMA Study Group (2004). A systematic approach to analyse health-related quality of life in multiple sclerosis: The GEDMA study. Mult. Scler..

[54] Forbes A., While A., Mathes L., Griffiths P. (2006). Health problems and health-related quality of life in people with multiple sclerosis. Clin. Rehabil..

[55] Dworzańska E., Mitosek-Szewczyk K., Stelmasiak Z. (2009). Zespół zmeczenia w stwardnieniu rozsianym [Fatigue in multiple sclerosis]. Neurol. Neurochir. Pol..

[56] Simmons R.D., Tribe K.L., McDonald E.A. (2010). Living with multiple sclerosis: Longitudinal changes in employment and the importance of symptom management. J. Neurol..

[57] Flensner G., Landtblom A.M., Söderhamn O., Ek A.C. (2013). Work capacity and health-related quality of life among individuals with multiple sclerosis reduced by fatigue: A cross-sectional study. BMC Public Health.

[58] Fricska-Nagy Z., Füvesi J., Rózsa C., Komoly S., Jakab G., Csépány T., Jobbágy Z., Lencsés G., Vécsei L., Bencsik K. (2016). The effects of fatigue, depression and the level of disability on the health-related quality of life of glatiramer acetate-treated relapsing-remitting patients with multiple sclerosis in Hungary. Mult. Scler. Relat. Disord..

[59] Calandri E., Graziano F., Borghi M., Bonino S. (2019). Young adults’ adjustment to a recent diagnosis of multiple sclerosis: The role of identity satisfaction and self-efficacy. Disabil. Health J..

[60] Wilski M., Tasiemski T. (2016). Illness perception, treatment beliefs, self-esteem, and self-efficacy as correlates of self-management in multiple sclerosis. Acta Neurol. Scand..

[61] Wassem R. (1992). Self-efficacy as a predictor of adjustment to multiple sclerosis. J. Neurosci. Nurs..

[62] Ziarko M. (2014). Zmaganie się ze Stresem Choroby Przewlekłej [Struggling with the Stress of a Chronic Disease].

[63] Mikula P., Nagyova I., Vitkova M., Szilasiova J. (2018). Management of multiple sclerosis: The role of coping self-efficacy and self-esteem. Psychol. Health Med..

[64] Kościelak R. (2010). Poczucie Umiejscowienia Kontroli i Przekonania o Własnej Skuteczności w Zdrowiu i w Chorobie [A Sense of Locating Control and Self-Efficacy in Health and Disease].

[65] Motl R.W., McAuley E., Snook E.M., Gliottoni R.C. (2009). Physical activity and quality of life in multiple sclerosis: Intermediary roles of disability, fatigue, mood, pain, self-efficacy and social support. Psychol. Health Med..

[66] Stuifbergen A.K., Seraphine A., Roberts G. (2000). An explanatory model of health promotion and quality of life in chronic disabling conditions. Nurs. Res..

[67] Motl R.W., McAuley E., Wynn D., Sandroff B., Suh Y. (2013). Physical activity, self-efficacy, and health-related quality of life in persons with multiple sclerosis: Analysis of associations between individual-level changes over one year. Qual. Life Res..

[68] Ferrier S., Dunlop N., Blanchard C. (2010). The role of outcome expectations and self-efficacy in explaining physical activity behaviors of individuals with multiple sclerosis. Behav. Med..

[69] Sikes E.M., Cederberg K.L., Baird J.F., Sandroff B.M., Motl R.W. (2019). Self-efficacy and walking performance across the lifespan among adults with multiple sclerosis. Neurodegener. Dis. Manag..

[70] Motl R.W., Balto J.M., Ensari I., Hubbard E.A. (2017). Self-efficacy and Walking Performance in Persons with Multiple Sclerosis. J. Neurol. Phys. Ther..

[71] Krok D., Gerymski R. (2019). Self-efficacy as a mediator of the relationship between meaning in life and subjective well-being in cardiac patients. Curr. Issues Personal. Psychol..

[72] Kossakowska M., Stefaniak T. (2017). Psychometric properties for the Polish version of the Brief Illness Perception Questionnaire (Brief IPQ). Health Psychol. Rep..

